# Peroxisome proliferator-activated receptors and their ligands: nutritional and clinical implications – a review

**DOI:** 10.1186/1475-2891-13-17

**Published:** 2014-02-14

**Authors:** Bogna Grygiel-Górniak

**Affiliations:** 1 Department of Bromatology and Human Nutrition, University of Medical Sciences, Poznan, Poland

## Abstract

Peroxisome proliferator-activated receptors are expressed in many tissues, including adipocytes, hepatocytes, muscles and endothelial cells; however, the affinity depends on the isoform of PPAR, and different distribution and expression profiles, which ultimately lead to different clinical outcomes. Because they play an important role in lipid and glucose homeostasis, they are called lipid and insulin sensors. Their actions are limited to specific tissue types and thus, reveal a characteristic influence on target cells. PPARα mainly influences fatty acid metabolism and its activation lowers lipid levels, while PPARγ is mostly involved in the regulation of the adipogenesis, energy balance, and lipid biosynthesis. PPARβ/δ participates in fatty acid oxidation, mostly in skeletal and cardiac muscles, but it also regulates blood glucose and cholesterol levels. Many natural and synthetic ligands influence the expression of these receptors. Synthetic ligands are widely used in the treatment of dyslipidemia (e.g. fibrates - PPARα activators) or in diabetes mellitus (e.g. thiazolidinediones - PPARγ agonists). New generation drugs - PPARα/γ dual agonists - reveal hypolipemic, hypotensive, antiatherogenic, anti-inflammatory and anticoagulant action while the overexpression of PPARβ/δ prevents the development of obesity and reduces lipid accumulation in cardiac cells, even during a high-fat diet. Precise data on the expression and function of natural PPAR agonists on glucose and lipid metabolism are still missing, mostly because the same ligand influences several receptors and a number of reports have provided conflicting results. To date, we know that PPARs have the capability to accommodate and bind a variety of natural and synthetic lipophilic acids, such as essential fatty acids, eicosanoids, phytanic acid and palmitoylethanolamide. A current understanding of the effects of PPARs, their molecular mechanisms and the role of these receptors in nutrition and therapeutic treatment are delineated in this paper.

## Introduction

**Figure 1:**
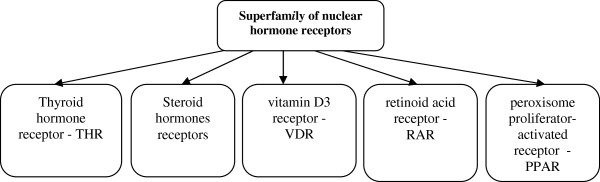
Superfamily of nuclear hormone receptors.

Peroxisome proliferator-activated receptors are ligand-activated transcription factors that regulate genes important in cell differentiation and various metabolic processes, especially lipid and glucose homeostasis. In molecular terms, PPARs represent a family of ligand-activated nuclear hormone receptors (NRs) belonging to the steroid receptor superfamily [1,2] (Figure 1). Examples of NRs include the receptors for thyroid hormones, retinoids, 1,25-dihydroxy-vitamin D3, steroid hormone receptors and a variety of other ligands. After interaction with the specific ligands, nuclear receptors are translocated to the nucleus, where they change their structure and regulate gene transcription [3-5].

### PPAR structure and function

**Figure 2:**
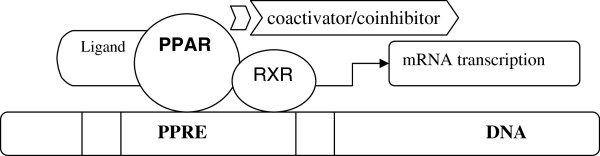
Mechanism of gene transcription by PPARs.

The three-dimensional structure of PPARs consists of a DNA binding domain in the N-terminus and a ligand binding domain (LBD) in the C-terminus. After interaction with agonists, PPARs are translocated to the nucleus and heterodimerize with another nuclear receptor - the retinoid X receptor (RXR) (Figure 2). The RXR forms a heterodimer with a number of other receptors (e.g., vitamin D or thyroid hormones). The specific DNA regions of target genes that bind with PPARs are termed peroxisome proliferator hormone response elements (PPREs) [1]. The PPREs are found in the promoters of PPAR responsive genes, such as the fatty acid-binding protein (aP2) [5]. In most cases, this process activates transcription of various genes involved in diverse physiological and pathophysiological processes.

The function of PPARs is modified by a number of co-activators and corepressors, the presence of which can either stimulate or inhibit receptor function, respectively [6]. Ligands that activate PPARγ-RXR cause an exchange of co-repressors for co-activators [7,8]. Human cells are characterized by a different availability of cofactors that depends on the type of cell and the association of specific cofactors to other genes [7,9,10].

### Types of PPARs and their tissue expression

**Figure 3:**
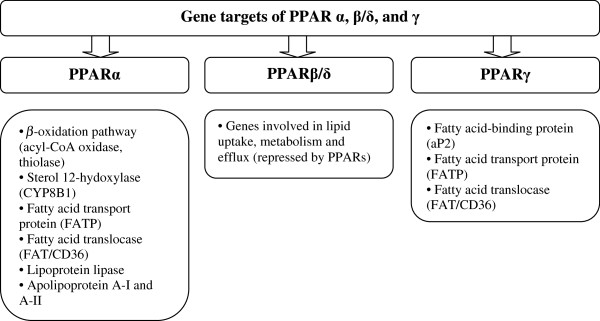
PPARs and their gene targets.

**Figure 4:**
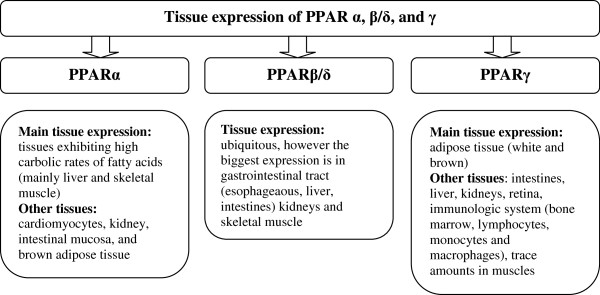
Expression of PPARs in specific tissues.

The family of peroxisome proliferation-activated receptors comprises three isoforms: PPARα, PPARβ/δ and PPARγ [1]. These three isotypes differ from each other in terms of their tissue distributions, ligand specificities and physiological roles. Each of them either activates or suppresses different genes with only partial overlap in activity (Figure 3) [5]. All isoforms participate in lipid homeostasis and glucose regulation (energy balance), and, until recently, their actions were thought to be limited to specific tissue types (Figure 4) [5,11]. PPARα is highly expressed in metabolically active tissues, such as liver, heart, skeletal muscle, intestinal mucosa and brown adipose tissue. This receptor is implicated in fatty acid metabolism and its activation lowers lipid levels [12-15].

PPARγ is expressed in white and brown adipose tissue, the large intestine and spleen. However, its expression is highest in adipocytes and it plays a key role in the regulation of adipogenesis, energy balance, and lipid biosynthesis [14,16-18]. This receptor also participates in lipoprotein metabolism and insulin sensitivity.

The least known isoform is PPARβ/δ, which has not been so intensely studied as PPARα and PPARγ. PPARβ/δ is expressed ubiquitously in virtually all tissues; however, it is particularly abundant in the liver, intestine, kidney, abdominal adipose tissue, and skeletal muscle, all of which are involved in lipid metabolism. It participates in fatty acid oxidation, mainly in skeletal and cardiac muscles, regulates blood cholesterol concentrations and glucose levels [1,13,19,20].

In conclusion, PPARα and PPARβ/δ mainly facilitate energy combustion, whereas PPARγ contributes to energy storage by enhancing adipogenesis [21].

### PPAR ligands

Many natural and synthetic agonists of PPARs are used in the treatment of glucose and lipid disorders. PPARs perform different activities, mainly via endogenous ligands produced in the metabolic pathways of fatty acids; and therefore, they are called lipid sensors. PPAR agonists have different properties and specificities for individual PPAR receptors, different absorption/distribution profiles, and distinctive gene expression profiles, which ultimately lead to different clinical outcomes [1,5,17,22,23].

**Figure 5:**
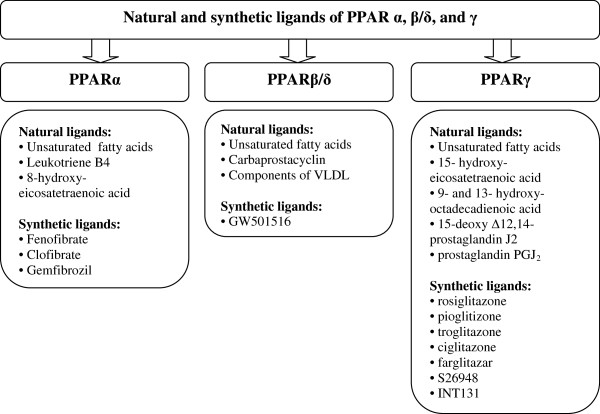
Natural and synthetic ligands of PPAR.

The characteristic feature of the PPAR ligand binding cavity is its size, which is 3–4 times larger than that of the other nuclear receptors. Thus, PPARs have the capability to accommodate and bind a variety of natural and synthetic lipophilic acids, such as essential fatty acids (EFA) (Figure 5). These acids act as PPAR agonists that transcript the genes involved in glucose and lipid homeostasis [12,22,24]. They include docosahexaenoic acid and eicosapentaenoic acid used in the prevention and treatment of cardiovascular and metabolic diseases [25]. Not only EFA but also eicosanoids are natural ligands of PPARs – e.g. leukotriene B_4_ stimulates PPARα, and prostaglandin PGJ_2_ activates PPARγ [22]. However, both EFA and eicosanoids are required in relatively high concentrations (approximately 100 μM) for PPAR activation [24]. Also, synthetic ligands are widely used in clinical practice – for example, fibrates (PPARα ligands) are recommended in the dyslipidemic state (hypertriglyceridemia) and thiazolidinediones (PPARγ agonists) are used in the treatment of diabetes mellitus [26-29].

### Functional role of PPARα

**Figure 6:**
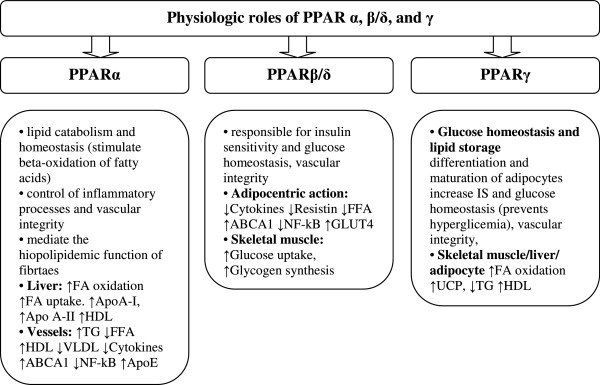
The role of PPARs (↑ - increase, ↓ - decrease).

**Figure 7:**
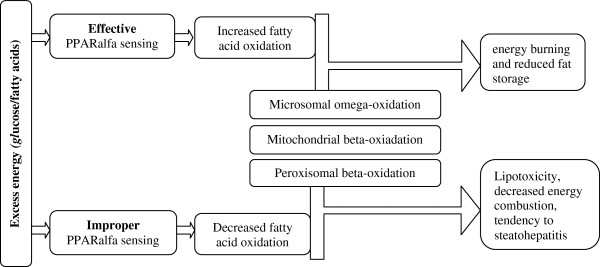
Consequence of PPARα activation.

As mentioned above, PPARα is expressed mainly in tissues with a high capacity for fatty acid oxidation, e.g. the liver, heart, and skeletal muscle. It also plays a role in glucose homeostasis and insulin resistance development (Figure 6) [29]. Natural or pharmacological ligands (fatty acids and fibrates, respectively) primarily control the expression of genes involved in lipid metabolism. If the concentration of fatty acids increases, PPARα is activated and uptakes oxidized forms of these acids [30,31]. Oxidation of fatty acids is mainly present in the liver and it prevents steatosis in the case of starvation/fasting. During the influx of fatty acids, transcription of PPARα-regulated genes is stimulated and the oxidation systems (microsomal omega-oxidation system, and mitochondrial and peroxisomal beta-oxidation) are activated (Figure 7) [21,32]. This activation and increased PPARα sensing in the liver result in increased energy burning and reduced fat storage. Conversely, ineffective PPARα sensing or decreased fatty acid oxidation causes a reduction in energy burning that results in hepatic steatosis and steatohepatitis (especially during overnight or prolonged fasting) [32,33]. The diminished effectiveness of oxidation systems is caused by genetic or toxic factors (including drug related ones), and metabolic disturbances. In animal models, inefficient PPARα sensing (characteristic for PPARα^_/_^ mice) enables the oxidation of the influxed fatty acids and leads to severe hepatic steatosis development. Administration of PPARα agonists prevents these processes and even reverses hepatic fibrosis (in animal models) [34]. Thus, PPARα functions as a lipid sensor and it controls energy combustion. It also plays also a prominent role in the pathogenesis of fatty liver disease (FLD) and ligands of this receptor might be effective in the reduction of hepatic staetosis by increasing energy utilization [32,33].

### Natural agonists of PPARα

The natural ligands of PPARα are omega-3 fatty acids. These acids contain three essential elements for optimal binding: a polar head group (a carboxylic group in the molecule of docosahexaenoic acid – DHA and eicosapentaenoic acid – EPA), a linker region (their long chains), and a hydrophobic tail [16]. Because omega-3 fatty acids are highly polyunsaturated, they readily undergo oxidation and stimulate PPARs. Sethi et al. proved that not only native EPA but also oxidized EPA modestly activates PPARα. Moreover, oxidized EPA, much more than native EPA, stimulates this receptor in endothelial cells. Therefore, it is suspected that oxidation of the omega-3 fatty acids converts them into a stronger PPARα agonist [30]. Similarly, oxidation of LDL transforms them into potent stimulators of PPARα in endothelial cells [31]. Thus, the possibility of lipid oxidation may be one of the first steps involved in the generation of efficient PPARα agonists.

In addition to PPAR activation, EPA and DHA have many beneficial health effects which are not characteristic for PPAR ligands. They reduce the risk of coronary heart disease, hypertension, primary heart attack, rheumatoid arthritis and play an important role in the development of attention-deficit/hyperactivity disorder [25]. These beneficial effects are not observed with other typical PPARs ligands; however, EPA and DHA do not show a number of PPAR effects, such as decreasing insulin resistance, that probably occur due to dissociation between n-3 polyunsaturated fatty acids and lipid metabolism in the hyperglycemic state [35].

Omega-3 fatty acids also reveal an anti-inflammatory effect that results from the inhibition of their own oxidation caused by activated NF-κB in a PPARα-dependent pathway [14,16]. In addition, PPARα mediates the anti-inflammatory actions of palmitoylethanolamide, the naturally occurring amide of palmitic acid and ethanolamine [15].

### Synthetic agonists of PPARα

Synthetic PPARα ligands such as fibrates (e.g. clofibrate, fenofibrate, and bezafibrate) decrease the triglyceride-rich lipoproteins in serum through an increase in the gene expression involved in fatty acid-β-oxidation and a decrease in apolipoprotein C-III gene expression [26,29]. They are widely used in the pharmacological treatment of hypertriglyceridemia. After activation by fibrates, PPARα receptors connect with the 9-cis retinoic acid receptor and then bind to peroxisome proliferator response elements. Fibrates not only reveal a triglyceride-lowering effect, but also increase HDL cholesterol (partly by increasing apolipoprotein A-I and A-II gene expression). Their activity results in decreasing systemic availability of fatty acids and a lowering of fatty acid uptake in muscles. They also increase insulin sensitization and reduce plasma glucose levels [12,26,29]. In consequence, they may slow arteriosclerosis progression and reduce cardiovascular events [36]. However, two large-scale studies into fibrates (the FIELDstudy and the ACCORDlipid arm) in patients with diabetes did not show any reduction in fatal cardiovascular incidences or non-fatal myocardial infarction and stroke compared with simvastatin alone [37,38]. It must also be underlined that fibrate intake may cause an increase in serum creatinine levels (via a decrease in tubular secretion, reduced clearance and possibly increased production of creatinine) [39-41]. However, long-term studies have shown a slowing of the decline in renal function [37-39].

Active metabolites of fibrates, such as clofibric acid and fenofibric acid, are dual activators of PPARα and PPARγ, with about a 10-fold selectivity for PPARγ. The effect of bezafibrate, another compound from this group, is wider because it activates all 3 PPAR subtypes (α, γ and δ) at comparable doses to other fibrates [1,5]. Therefore, bezafibrate is considered a panagonist for all 3 PPAR isoforms with the potential to directly improve insulin sensitization via PPARγ activation.

### Functional role of PPARβ/δ

PPARβ/δ (referred to also as PPARδ, PPARβ, hNUC1 or FAAR) plays a key role in lipid and cholesterol metabolism. It is implicated in fatty acid oxidation, improving lipid profiles and reducing adiposity, which in consequence prevents obesity development [19,42]. In animals, PPARβ/δ serves as a regulator of fat consumption. PPARβ/δ-deficient mice challenged with a high-fat diet showed reduced energy uncoupling and were prone to obesity, whereas PPARβ/δ activation led to resistance to nutritionally (induced by high-fat diet) or genetically triggered obesity (in genetically modified mice) [19].

Decreased PPARβ/δ expression is also observed in cardiac muscle during the hyperglycemic state in diabetes mellitus [43,44]. Conversely, overexpression of this receptor in cardiac cells diminishes lipid accumulation in the presence of a high fat diet, and increases glucose metabolism. In consequence, the heart is protected against ischemia-reperfusion injury, suggesting that activation of this receptor might be useful in diabetic cardiomyopathy [45].

Also beneficial is PPARβ/δ activation in adipocytes and skeletal muscles that results in fatty acid oxidation and utilization (studies in vitro) [19]. Thus, PPARβ/δ might be considered a potential target in the treatment of obesity and obesity related disorders. This receptor is also a mediator of embryo implantation and cancer development [42,46]. Its high expression in the large intestine has been implicated in the development of colon cancer [11,47]. In this process, PPARβ/δ was stimulated by arachidonic acid, which resulted in the upregulation of cyclooxygenase (COX)-2 and the overproduction of prostaglandin (PG)E2 – an activator of colon cancer cells. Similarly, activation of PPARβ/δ also stimulated the cell line proliferation of human breast and prostate cancer [20].

### Agonists of PPARβ/δ

Dual agonists of PPARβ/δ improve insulin sensitivity in both murine models and in humans. Thus, they might be considered a potential target in the treatment of obesity and obesity associated disorders. Only bezafibrate, a traditional PPARα agonist, seems to be a safe pan-agonist for all PPAR isotypes, but it has relatively low potency and low affinity for PPARβ/δ [48].

### Clinical and nutritional role of PPARγ

PPARγ has attracted significant scientific and clinical interest because of its role in macronutrient metabolism. It is a target of the synthetic insulin sensitizers – thiazolidinediones - used in the treatment of type 2 diabetes mellitus. This receptor is abundantly expressed in adipose tissue where it plays a central role in adipogenesis and appears to be primarily involved in the regulation of lipid metabolism. Because the PPARγ gene has separate promoters and 5’exons, it results in three mRNAs: PPARγ1, PPARγ2, and PPARγ3. Proteins produced from PPARγ1 and PPARγ3 mRNAs are identical, whereas the product of PPARγ2 contains an additional NH_2_-terminal region composed of 30 amino acids. All PPARγ isoforms play an important role in adipocyte differentiation and glucose metabolism; however, their expression is different. The PPARγ1 isoform is expressed in nearly all cells, while PPARγ2 is limited mainly to adipose tissue. Nevertheless, PPARγ2 is a more potent transcription activator [8].

Both forms of PPARγ1 and PPARγ2 are essential for the development of adipose tissue and the control of insulin sensitivity. However, PPARγ2 is the isoform regulated in response to nutrient intake and obesity [9,10]. The study of Medina-Gomez et al. based on an animal model proved that removing PPARγ2 from genetically obese POKO mice decreases the fat accumulation in adipocytes compared to normal obese mice on the same diet. This study showed that the PPARγ2 isoform prevents lipotoxicity in different mechanisms including promotion of adipose tissue expansion, the augmentation of lipid-buffering capacity in peripheral organs (liver, muscle, and beta cells of pancreas), and the proliferative response of β-cells to insulin resistance [18].

Adipose PPARγ protects non-adipose tissues against excessive lipid overload and maintains normal organ function (liver, skeletal muscle). Activated PPARγ in adipocytes guarantees a balanced and adequate secretion of adipocytokines (adiponectin and leptin) that are mediators of insulin action in peripheral tissues. In consequence, the insulin sensitivity of the whole body is maintained [49]. Apart from this adipogenetic activity, PPARγ is also important in lipid metabolism, and it regulates the genes participating in the release, transport, and storage of fatty acids such as lipoprotein lipase (LPL) and the fatty acid transporter CD36 [9,17,18]. PPARγ is the potent function modulator not only found in adipose tissue but also in endothelial cells and vascular smooth muscle cells. In endothelial cells it regulates targets relevant to inflammation and atherosclerosis [50].

Despite controlling lipid metabolism, PPARγ also participates in the regulation of cancer development. Their agonists inhibit or promote cancer growth depending on cellular conditions and the stimulated signaling pathway (anti-proliferative and apoptotic) [8,31]. They influence tumor-associated macrophages and tumor vasculature and significantly attenuate tumor progression [23]. These data suggested that PPARγ ligands may become new convenient therapeutic modifiers targeting simultaneously tumors and their microenvironment [51].

### Natural agonists of PPARγ

The selective PPARγ modulators are often called SPARMs by analogy to selective estrogen receptor modulators (SERMs). The distinct actions of SPARMs depend on the cellular context and on different receptor conformations, resulting in diverse gene interactions [52].

Selected fatty acids are considered natural modulators of PPARγ; however, their connection with the receptor does not always lead to PPAR activation and target gene transcription. Activation of PPARγ by natural ligands such as PUFAs (mainly docosahexaenoic acid and eicosapentaenoic acid) results in a functional response in the tumor cells. Many studies have provided evidence that DHA inhibits the tumor development through activation of PPARγ (e.g. the growth of human lung cancer cells) [53]. If DHA is delivered to breast cancer cells by albumin or by enriched LDL with n-3 PUFAs, it inhibits the proliferation of these cells and stimulates their apoptosis [54-56]. Syndecan-1 (the heparan sulfate proteoglycan) - the activating factor of apoptosis - participates in this process. After the stimulation of PPARγ by DHA, the transcriptional upregulation of the syndecan-1 target gene is present [56]. In animal studies long-chain monounsaturated fatty acids (LC-MUFAs) with chain lengths longer than 18 (i.e., C20:1 and C22:1 isomers combined) may ameliorate obesity-related metabolic dysfunction through increased expression of PPARγ and decreased inflammatory marker expression in white adipose tissue [57]. In in vitro studies, activation of PPARα and PPARβ/δ in human cell lines of breast cancer stimulated cell proliferation [58,59], whereas ligands for PPARγ inhibited this process [20].

Apart from polyunsaturated fatty acids, phytanic acid is also a natural PPARγ agonist in the human diet that reveals a similar activity to omega-3 PUFA and increases glucose uptake and insulin sensitivity; however, with less capacity to differentiate adipocytes [60].

### PPARγ pharmacological agonists

PPARγ is a regulator of lipid and glucose metabolism and therefore its synthetic ligands such as glitazones – the derivatives of thiazolidinediones (e.g., troglitazone, rosiglitazone and pioglitazone) - improve insulin and glucose parameters and increase whole body insulin sensitivity. Therefore, they are called insulin-sensitizing medications used in the treatment of diabetes [61]. They indirectly increase insulin-stimulated glucose uptake in adipocytes, hepatocytes and skeletal muscle cells [62,63]. The effects of pharmacological PPARγ activation by thiazolidinediones have been attributed, at least in part, to decreased free fatty acid (FFA) levels and increased lipid storage in adipose tissue, in which it is most highly expressed. In consequence, lipotoxicity in the muscles and liver is reduced. PPARγ agonists also have the ability to redistribute fat from visceral to subcutaneous depots and increase adiponectin and reduce tissue necrosis factors [63,64]. Moreover, rosiglitazone and pioglitazone are used in the treatment of patients with type 2 diabetes because they decrease hepatic glucose production and prolong pancreatic β-cell function by preventing apoptosis of β-cells [62,63]. They reduce fasting plasma glucose and glycated hemoglobin A1c (pioglitazone 15–45 mg/day, rosiglitazone 2–6 mg/twice a day) [65-67]. However, the influence of both thiazolidinediones on cardiovascular outcomes in patients with diabetes mellitus is different. The positive effect of pioglitazone was demonstrated in a PROactive study revealing 16% reduced cardiovascular complications in the main secondary endpoint (a composite of all-cause-mortality, non-fatal myocardial infarction and stroke) compared with placebo treatment [68]. Conversely, rosiglitazone was associated with significant increases in myocardial infarction and death from cardiovascular causes after only relatively short-term exposure [69] and the European Medicines Agency withdrew approval of this medication in 2010 due to these cardiovascular safety concerns [64]. The differences between thiazolidinediones are probably caused by their diverse effects on lipid sub-fractions [63]. Pioglitazone increases HDL cholesterol and decreases triglycerides and fasting plasma free fatty acids (without any influence on total cholesterol and LDL cholesterol) [64,70,71]. Rosiglitazone significantly augments HDL levels [64,70,71], total cholesterol and the LDL fraction [71].

In diabetes mellitus, long-term activation of PPARγ by thiazolidinediones not only reduces glycaemia and insulinemia but also attenuates vascular dysfunction [72]. PPARγ is expressed in vessels, specifically in vascular smooth muscle cells and endothelium. Recent studies suggest that activators of PPARγ not only modify metabolic disturbances but also protect vascular function in diabetes [72]. Using an animal model, Bagi et al. proved that short-term treatment of type 2 diabetic mice with rosiglitazone augmented NO-mediated flow-dependent dilations of coronary arterioles by reducing vascular superoxide production via a favorable alteration of oxidant/antioxidant enzyme activities [73]. PPARγ agonists also lower blood pressure and decrease circulating PAI-1 and CRP levels in patients with diabetes [27,28].

Apart from these positive effects, activation of PPARγ by glitazones attenuates systemic inflammation [3,4] and reduces tumor growth and angiogenesis. PPARγ activation by agonist RS5444 may inhibit anaplastic thyroid cancer growth [74]. Only troglitazone revealed tumor-promoting and pro-angiogenic properties – it promoted hepatic carcinogenesis and liposarcomas, and thus this agonist was rejected from the treatment [72,75].

Despite many beneficial features of glitazones (metabolic and anti-arteriosclerotic activity), they also exhibit side effects, such as weight gain, edema, bone fractures, heart failure and increased risk of myocardial infarctions, which have limited the use of these drugs in diabetic patients with high lipid levels [76]. Fortunately, new selective PPAR*γ* modulators are currently in development (such as S26948 [77] and INT131 [78]) and these should stimulate glucose metabolic pathways and minimize the side effects of full PPAR-*γ* agonists.

### PPARα/γ dual agonists

The synthesis of new drug generation - PPARα/γ dual agonists - connecting positive influences on both lipid and glucose metabolism has been recently developed as a response to the treatment challenge of co-existing type 2 diabetes mellitus with dyslipidemia. These double agonists not only have anti-diabetic capacity but also reduce arteriosclerosis development. They also exhibit anti-inflammatory and anticoagulant action, improve endothelial function, decrease plasma free fatty acids and lower blood pressure revealing beneficial effects on the vasculature. However, recent studies show that PPARα/γ dual agonists, similar to glitazones, exhibit the same side effects; for example, weight gain and edema [48,79]. None of them may be safely used in clinical treatment because of the increased risk of bladder cancer and hyperplasia (ragaglitazar and naveglitazar) or [80], renal dysfunction (tesaglitazar) [81] and increased cardiovascular risk (muraglitazar) [82]. A recent study (currently in phase III trials) of aleglitazar decreased HbA1c and revealed beneficial effects on lipid profiles reducing triglyceride and LDL, and increasing HDL cholesterol. It changes the atherogenic small dense LDL particles into larger LDL molecules [108] and in consequence reduces inflammatory/cardiovascular risk [83]. This thiazolidinedione with a balanced affinity for both *α* and *γ* receptor subtypes is very promising; however, further studies are needed.

## Conclusions

Considering the wide range of actions on glucose, lipid metabolism and cell proliferation/apoptosis, PPARs and their modulators are suggested for the treatment of metabolic disorders such as hyperglycaemia, dyslipidemia and atherosclerosis. The prevention and treatment of both lipid and glucose profile disorders should consider the potency and affinity of selective PPARs and their potential cancerogenic influences. Therefore, natural compounds and their close derivatives are being targeted as future drugs against metabolic diseases. Even though early preclinical data are very promising, it is necessary to evaluate the clinical properties of new PPAR agonists and their influence on patient health.

## Competing interests

The author declares that he has no competing interests.
